# Phase Shifting Capacity of the Circadian Pacemaker Determined by the SCN Neuronal Network Organization

**DOI:** 10.1371/journal.pone.0004976

**Published:** 2009-03-23

**Authors:** Henk Tjebbe vanderLeest, Jos H. T. Rohling, Stephan Michel, Johanna H. Meijer

**Affiliations:** 1 Department of Molecular Cell Biology, Laboratory for Neurophysiology, Leiden University Medical Center, Leiden, The Netherlands; 2 Leiden Institute of Advanced Computer Science, Leiden University, Leiden, The Netherlands; Vanderbilt University, United States of America

## Abstract

**Background:**

In mammals, a major circadian pacemaker that drives daily rhythms is located in the suprachiasmatic nuclei (SCN), at the base of the hypothalamus. The SCN receive direct light input via the retino-hypothalamic tract. Light during the early night induces phase delays of circadian rhythms while during the late night it leads to phase advances. The effects of light on the circadian system are strongly dependent on the photoperiod to which animals are exposed. An explanation for this phenomenon is currently lacking.

**Methodology and Principal Findings:**

We recorded running wheel activity in C57 mice and observed large amplitude phase shifts in short photoperiods and small shifts in long photoperiods. We investigated whether these different light responses under short and long days are expressed within the SCN by electrophysiological recordings of electrical impulse frequency in SCN slices. Application of N-methyl-D-aspartate (NMDA) induced sustained increments in electrical activity that were not significantly different in the slices from long and short photoperiods. These responses led to large phase shifts in slices from short days and small phase shifts in slices from long days. An analysis of neuronal subpopulation activity revealed that in short days the amplitude of the rhythm was larger than in long days.

**Conclusions:**

The data indicate that the photoperiodic dependent phase responses are intrinsic to the SCN. In contrast to earlier predictions from limit cycle theory, we observed large phase shifting responses in high amplitude rhythms in slices from short days, and small shifts in low amplitude rhythms in slices from long days. We conclude that the photoperiodic dependent phase responses are determined by the SCN and propose that synchronization among SCN neurons enhances the phase shifting capacity of the circadian system.

## Introduction

The daily revolution of the earth causes 24 hour cycles in the environmental conditions, while the annual cycle of the earth moving around the sun brings about seasonal changes. Many organisms possess an endogenous 24 hour or ‘circadian’ clock, which allows them to anticipate and adapt to the daily and annual environmental changes [1]. In mammals, a major pacemaker for circadian rhythms is located in the suprachiasmatic nuclei (SCN) of the anterior hypothalamus [2]. The ability of the SCN to generate circadian rhythms is present at the single cell level and is explained by a molecular feedback loop in which protein products of period and cryptochrome clock genes inhibit their own transcription [3], [4]. The SCN control circadian rhythms in molecular, endocrine and physiological functions, as well as in behavior [5]. Besides their role as a daily clock, the SCN are an integral part of the photoperiodic time measurement system and convey day length information to the pineal gland and other parts of the central nervous system [6]–[8].

The SCN are synchronized to the environmental light-dark cycle via the retina. Light information reaches the SCN directly via the retino-hypothalamic tract, which innervates the SCN with glutamate and pituitary adenylate cyclase activating peptide containing fibers [9]. Synchronization to the environmental light-dark cycle is based on a time-dependent responsiveness of the SCN to light, which is most easily demonstrated in “perturbation experiments” in which animals are kept in constant darkness and subjected to discrete pulses of light. Light pulses presented during the early night induce phase delays of the rhythm, while at the end of the night, they induce advances. The characteristic phase dependent light responsiveness is a prerequisite for animals to entrain to the environmental cycle, and is a common property of many organisms [10].

The maximum advancing and delaying capacity depends strongly on the photoperiod to which animals are exposed [10]–[12]. This finding has received surprisingly little attention, given the robustness of the photoperiodic modulation and potential functional significance. For instance in the hamster, the phase shifting effects of a 15 min light pulse on behavioral activity rhythms are about 2–3 fold larger in short winter days than they are in long summer days [10]. One possibility is that increased light exposure in long days desensitizes the system to light at the level of the retina [11]. Recently, it has become known that the organization of the SCN shows plasticity under influence of changes in day length [13]–[18]. The variation in light response over the seasons could therefore also result from different response properties brought about by plasticity within the SCN itself. We performed behavioral and electrophysiological experiments and found evidence that the phase shifting magnitude is determined by the SCN. The large phase shifts observed in high amplitude rhythms in short days versus the small shifts in long days leads us to propose that synchronization among individual oscillator components enhance the phase resetting capacity.

## Results and Discussion

**Figure 1:**
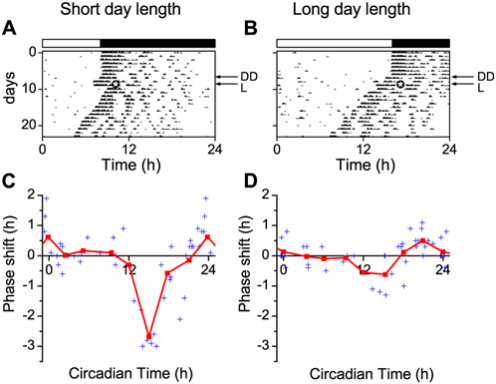
Phase shifts of wheel running behavior in mice induced by 30 minutes light pulses. (A, B) Examples of wheel running actograms from animals kept in short (A) and long photoperiods (B). The actograms show the wheel running activity of the mice over the 24 h day. Consecutive days are plotted on successive lines. The top bar indicates the light-dark schedule before transfer to continuous darkness (DD, indicated with an arrow). A light pulse was given on day four in DD (L, indicated with an arrow), 3 hours after activity onset (indicated by 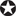 in the actogram). Activity onset was defined as circadian time 12. Phase response plots to 30 minute light pulses in short (C) and long (D) photoperiod. Phase responses are plotted as a function of the circadian time of the light pulse. Individual phase shifts are indicated by a plus symbol. The results were grouped in 3 h bins centered at CT 0, 3, 6, 9, 12, 15, 18 and 21. The average phase responses of the light pulses are indicated by squares and connected with a solid line. The time of maximal delay is at CT 15 for both long and short photoperiods and is significantly different between both day lengths (p<0.001). The large magnitude of the delays observed in short days is consistent with other studies [10], [11].

We performed behavioral experiments to establish phase shifting effects of light under long and short photoperiods. Running wheel activity was recorded from C57 mice kept in short and long day length (light∶dark 8 h∶16 h and 16 h∶8 h). After at least 30 days of entrainment to the light-dark cycle, the animals remained in continuous darkness for 3 days (Figure 1). On day 4 in darkness, the animals received a saturating 30 min white light pulse which was aimed at different phases of the circadian cycle. The onset of behavioral activity was used as a marker of circadian phase, and defined as circadian time 12 (CT 12). Maximum delays were observed for pulses given 3 hours after activity onset in both animals from short days (shift: −2.68±0.19 h, n = 6) and animals from long days (shift: −0.62±0.28 h, n = 5). The magnitude of the delays was however significantly larger for animals in short days (p<0.001). Light pulses towards the end of the night produced small phase advances which were not significantly different between the groups (short day advance: 0.61±0.26 h, n = 8; long day advance 0.50±0.11 h, n = 9; p>0.6).

To investigate whether the small phase delays in long days may have resulted from a decrease in photic sensitivity of the circadian system as a consequence of higher photon stimulation during entrainment, we reinvestigated the phase delaying effects of light in short days, and doubled the amount of photons to which we exposed the animals during the entrainment period. Thus, the short day animals received the same amount of photons as the long day animals, but now distributed over 8 instead of 16 hours. We found that the phase delaying effects of light were large (shift: −2.95±0.19 h, n = 8) and not different from those observed in short days under normal light conditions (p>0.1). The shifts were, however still significantly larger than those observed in long days (p<0.001). The results indicate that the difference in shift between long and short day animals is not attributable to an increment in photon stimulation during entrainment to long days.

**Figure 2:**
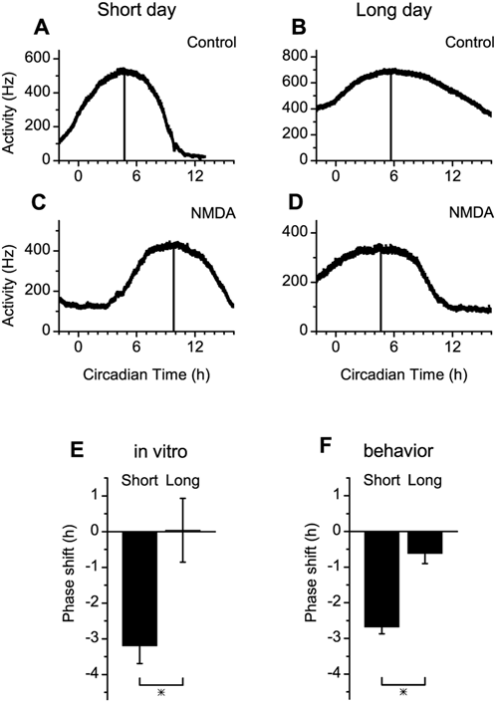
Phase shifts of multiunit electrical activity rhythms in brain slices from mice kept on a short and long photoperiod. Examples of extracellular multiunit recordings from the SCN in mice kept on a short photoperiod (A, C) and on a long photoperiod (B, D). Action potentials were counted in 10 s bins, and are plotted as a function of circadian time, determined by activity onsets from the mice prior to slice preparation. NMDA pulses were given 3 hours after the activity onset (CT 15), on the first cycle in vitro, in slices from both short (C) and long (D) day animals. In slices obtained from short day animals these pulses induced a delay in the peak time of the rhythm on the day following the application. Peak times are indicated by a vertical line. (E) Delays obtained at CT 15 from short day animals were significantly larger than delays obtained from long day animals. The magnitude of the delay after an NMDA pulse at CT 15 was significantly different between day lengths (p<0.01). (F) The magnitude of the behavioral delay was not different from the delay observed in vitro, for both day lengths (short day in vitro vs. behavior p>0.3, long day in vitro vs. behavior, p>0.4).

To investigate whether the difference in the magnitude of the phase shift in long and short days is retained in the SCN in vitro we kept animals under long and short day length and prepared hypothalamic slices containing the SCN on the third day after release in constant darkness (Figure 2). We recorded electrical impulse frequency in the SCN by stationary electrodes and applied NMDA pulses (10 µM, 30 min duration) by switching from regular artificial cerebrospinal fluid (ACSF) to NMDA containing ACSF. The NMDA receptor is of crucial importance in mediating phase shifting by light and application of the glutamate receptor agonist NMDA to brain slices in vitro generates phase shifts of the circadian rhythm resembling photic phase responses [19]–[21]. The timing of the NMDA pulse was based on the extrapolated behavioral activity of the animal before slice preparation, and was aimed 3 hours after activity onset, where the largest shifts in behavior were observed in both photoperiods.

**Figure 3:**
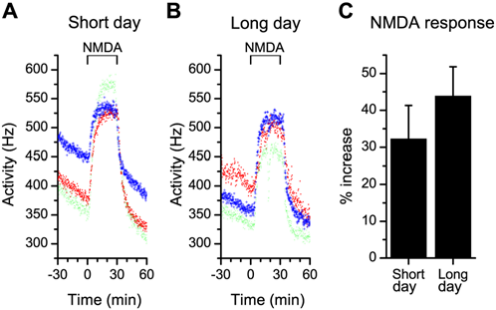
Acute responses in multiunit electrical activity to NMDA application at CT15. (A, B) NMDA application (10 µM) at CT 15 induced an increase in firing rate that was recorded by extracellular multiunit electrodes. The magnitude of the NMDA response is similar in slices from long and short day animals and in both photoperiods, a plateau was reached during the application. (C) The magnitude of the acute response to NMDA, measured as the relative increase in discharge rate, was not different between day lengths (p>0.3).

NMDA induced a sustained increment in SCN electrical discharge in slices from both photoperiods (Figure 3). The relative increase in electrical activity was 32.2±9.1% (n = 5) of baseline discharge in short days and 43.9±8.0% (n = 5) of baseline discharge in long days. No significant differences in responsiveness to NMDA were observed (p>0.1). Despite the similarity in acute NMDA responses, the resulting phase shifts were significantly larger in short days (−3.2±0.50 h, 6 control and 5 experimental slices) compared to long days (0.0±0.89 h, 6 control and 5 experimental slices; p<0.006; Figure 2). We also calculated the phase shift based on a secondary phase marker, the time of half maximum value on the rising slope of the electrical discharge peak. With this phase marker we found the same difference in phase shift between long and short day length, indicating the robustness of the measured differences in phase shift (difference in phase shift between day lengths: 3.2±0.86 h; p<0.002).

The data indicate that the phase shifting capacity of the circadian system under long and short photoperiods is determined by the SCN itself. The absence of a difference in the magnitude of the NMDA response underscores this interpretation and shows that the same increase in neuronal activity of the SCN results in a different phase shifting response.

In an additional series of experiments we treated 7 slices from long day animals with 25 µM NMDA at CT 15. The acute increase in electrical activity in response to 25 µM NMDA was 123±16.0% as compared to baseline, which is three times larger than the response to 10 µM NMDA (p<0.003). The phase-shifts in electrical activity rhythms were however not significantly different from untreated or 10 µM NMDA treated slices (shift: −0.29±0.72 h; p>0.68 in both cases). These results show that for long day length, the phase shifting response is not enhanced by an increment of the pharmacological stimulus. The preservation of the small shifts in slices from long days indicates an intrinsic incapability of the SCN to shift in long photoperiods.

The question arises what mechanism in the SCN underlies the photoperiodic modulation of the phase shifting capacity. Recently it has become clear that photoperiodic encoding by the SCN [7], [22] is accomplished through a reconfiguration of cellular activity patterns [13], [16]–[18], [23]. In long days, the activity patterns of single SCN neurons are spread in phase, rendering a broad population activity pattern, while in short days, the neurons oscillate highly in phase, which yields a composite waveform with a narrow peak [13], [17]. Molecular studies have shown regional differences in gene expression patterns within the SCN that increase in long days and decrease in short days [16], [18], [23].

**Figure 4:**
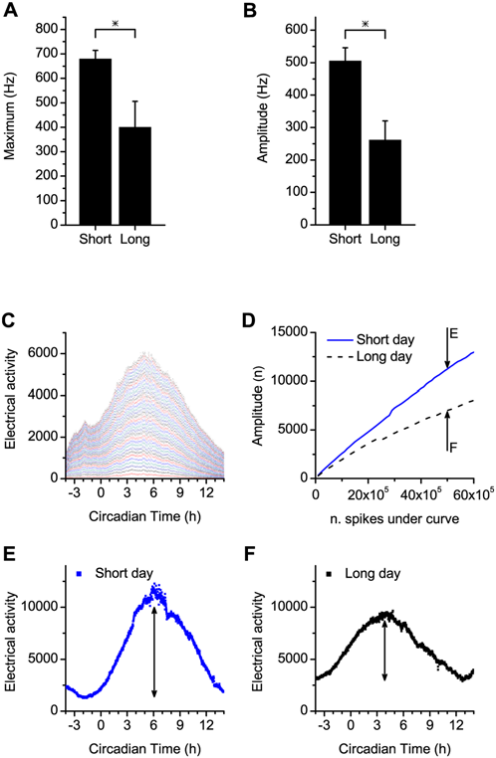
Amplitude of the electrical activity peak in short and long days. (A) Maximal firing frequency in multiunit activity, recorded in slices from animals maintained in short and long days were significantly different (p<0.05). (B) Amplitudes of the multiunit activity rhythm, defined as the difference between maximal and minimum firing level, were significantly different between the short and long day groups (p<0.01). (C) An analysis was performed, in which the total number of action potentials contributing to the electrical activity pattern was determined. This allowed for a comparison of rhythm amplitude between the experiments for multiple sizes of subpopulations. Each line represents an increase over the lower line of a total number of 10^5^ action potentials included in the recording. Action potentials were counted in 60 s bins. (D) Amplitude of electrical activity rhythms in subpopulations with a selected number of action potentials included in the recording. The amplitude of the electrical activity in the short day group is larger than the amplitude in the long day group for recordings with an equal number of action potentials. For 50×10^5^ action potentials (indicated by arrows), examples of subpopulation electrical activity patterns are indicated in E and F. The difference in amplitude between long and short days exists for any number of action potentials contributing to the curve, and thus, for any subpopulation size. (E, F) Examples of electrical activity patterns obtained in short (E) and long (F) days, with the same total number of action potentials (50×10^5^) making up the electrical activity pattern.

Theoretically, it follows from such a working mechanism, that the amplitude of the SCN rhythm in short days is larger than the amplitude in long days. That is, when neurons overlap in phase in short days, the maximum activity of each neuron will be at similar phases, leading to a high frequency in multiunit activity due to the summed activity of overlapping units during the peak, while during the trough, non-overlapping units lead to low activity [15]. We measured the frequency of the multiunit activity of SCN neurons in long and short day slices and found that indeed, the maximum discharge levels are higher in short day animals (Figure 4). A general assumption in the field of circadian rhythm research is that high amplitude rhythms are more difficult to shift than low amplitude rhythms [24], which stands in contrast to our present findings. To critically test the observed amplitude differences, we analyzed the amplitude under long and short days in more detail, by an off-line analysis of subpopulation activity. To test if the amplitude differences are inherent to a change in photoperiod and are not influenced by threshold settings, we analyzed the amplitude at different threshold settings, reflecting the activity of different sizes of populations of SCN neurons (c.f. [13], [17]). In this analysis, we could reliably compare subpopulation activity rhythms, with an equal number of action potentials contributing to the circadian waveform. The results showed that in short days, the amplitude of the rhythm was larger than in long days for any given number of spikes in the recording (Figure 4D).

These findings are in contrast to the general assumption that the magnitude of a phase shift is inversely related to the amplitude of the rhythm, i.e. that it is more difficult to shift high amplitude rhythms than low amplitude rhythms. This assumption is based on the theory of limit cycle oscillators, where a perturbation of similar strength changes the phase of an oscillator with low amplitude more than one with higher amplitude, because the perturbation represents a larger fraction of the radius of the circle [25], [26]. The question is how to explain our current findings.

It could be argued, that in long day length, with a wide phase distribution, the neurons have a more diverse phase shifting response to a light input signal, while in short day length, with a narrow phase distribution, neurons may respond more coherent, resulting in a larger overall shift. Simulations were performed in which single unit PRCs were distributed over the light dark cycle according to experimentally obtained distributions of SCN subpopulations (see Text S1 and Figure S1). We used type 0 and type 1 PRCs as well as the single unit PRC described by Ukai et al. [27].The simulations showed that the amplitude of the long day PRC is considerably smaller than the short day PRC, irrespective of the shape of the single unit phase response curve that was used in the simulations. The results from our simulations accord with our behavioral and electrophysiological results (Figures S2 and S3). Recent studies have shown that a phase resetting light pulse alters the phase relation between oscillating fibroblasts and SCN neurons [27], [28]. Our results show that, vice versa, the phase relation between neurons determines the phase response of the ensemble. Together the data indicate a close relation between phase resetting behavior and the synchrony among oscillating cells.

While our data suggest that the phase relationship among oscillators determines the response to a shifting pulse, we acknowledge that other mechanisms cannot be ruled out. Our explanation is parsimonious, however, as two major aspects of photoperiodic encoding by the SCN, namely changes in circadian waveform and changes in light resetting properties, can be explained by changes in phase distribution within the SCN.

In summary, our findings indicate that the phase shifting capacity of the SCN expressed in long and short day length is retained in the SCN in vitro, offering an attractive model for future investigation. Our data also show that the inverse relation between the phase shifting capacity and the amplitude of the neural activity rhythm may not hold for neuronal networks in which neurons oscillate with different phases. We have shown that such networks respond in fact opposite, and show a maximum phase shifting capacity when the rhythm amplitude is large, and a smaller response when the amplitude is low. The data provide a clear example that neuronal networks are governed by different rules than single cell oscillators. To predict the phase response characteristics of the SCN network, we have taken into account the phase distribution among the single cell oscillators. We realize that a more accurate prediction of the properties of the network can be obtained when the interactions between the single cell oscillators are incorporated [29]–[31]. In the past few years a number of synchronizing agents have been proposed such as VIP, GABA, and gap junctions [32]–[37], and it would be interesting to determine their role in photoperiodically induced changes in the phase resetting properties of the SCN. Our findings may be relevant not only for the photoperiodic modulation of the phase shifting capacity of the circadian system, but may have broader implications and be relevant also to observations of reduced light responsiveness and reduced circadian rhythm amplitude in the elderly.

## Materials and Methods

### Ethics Statement

All experiments were performed in accordance to animal welfare law and with the approval of the Animal Experiments Ethical Committee of the Leiden University Medical Center.

### Behavioral Experiments

Mice (C57BL6) were kept under long (16 h light, 8 h dark) and short (8 h light, 16 h dark) photoperiods for at least 30 days in clear plastic cages equipped with a running wheel. The animal compartments are light tight and illuminated by a single white fluorescent “true light” bulb with a diffuse glass plate in front. The light intensity at the bottom of the cage was ∼180 lux. Running wheel activity was recorded with Actimetrics software and the onset of activity was determined as circadian time 12 (CT 12). After at least 30 days in the light regime, the animals were released into constant darkness (DD). On day 4 in DD, the animals received a 30 min white light pulse (180 lux) at a specific CT. We have previously shown that after 4 days in constant darkness, photoperiodic effects on behavioral activity and on SCN waveform are still fully present [17]. For each animal in the compartment, the average onset of activity was calculated and the CT of the light pulse was determined. Running wheel activity was recorded for another 14 days after the light pulse. The phase shifts were calculated by comparing activity onset in DD before and after the light pulse. The circadian times at which the light pulses were given were binned in 3 h intervals.

### In Vitro Experiments

Animals were housed under long and short photoperiods, as described before, for at least 30 days. Prior to the in vitro experiment, the animals were transferred to a dark compartment for 3 days. Onset of wheel running activity was determined over these 3 days and decapitation and subsequent dissection of the brain was performed at the end of the resting period of the animal (CT 12). Slices of 400 µm were prepared with a chopper and were transferred to a laminar flow chamber that was perfused with warmed (35°C) ACSF within 6 min after decapitation [13]. The pH was controlled by a bicarbonate buffer in the ACSF and was maintained by gassing the solution and blowing warmed humidified O_2_ (95%) and CO_2_ (5%) over the slice. The slice was kept submerged and was stabilized with an insulated tungsten fork.

The slices settled in the recording chamber for ∼1 h before electrode placement. Action potentials were recorded with 90% platinum 10% iridium 75 µm electrodes, amplified 10 k times and bandpass filtered (300 Hz low, 3 kHz high). The action potentials crossing a preset threshold well above noise (∼5 µV) were counted electronically in 10 s bins by a computer running custom made software. Time of occurrence and amplitudes of action potentials were digitized by a CED 1401 and stored for off-line analysis. To induce a phase shift, the recording chamber was perfused with ACSF containing 10 or 25 µM N-methyl-D-aspartate (NMDA) for 30 min. The timing of the NMDA application was in accordance with the light pulse presentation in the behavioral experiments: The slices were prepared on day 3 in DD and on the fourth night in DD the NMDA pulse was applied at CT 15. The estimation of CT 15 was done one the basis of the activity onsets of the animals in DD, on the days preceding the preparation of the slice.

### Data Analysis

Electrophysiological data was analyzed in MATLAB using custom made software as described earlier [17]. The time of maximum activity was used as marker for the phase of the SCN and was determined on the first peak in multiunit activity, both for control and experimental slices. Multiunit recordings of at least 24 h, that expressed a clear peak in multiunit activity, were moderately smoothed using a least squares algorithm [38] and peak time, half maximum values and amplitude were determined in these smoothed recordings.

For a more detailed analysis of rhythm amplitude, we used the stored times of the occurrence and amplitudes of the action potentials. This analysis allows for an off-line selection of the size of the population of neurons that contributes to the electrical activity rhythm, through a selection of voltage thresholds (see also Schaap et al., 2003 [13] and VanderLeest et al., 2007 [17]). In this way, we could describe the circadian activity pattern of larger or smaller subpopulations of SCN neurons. This analysis was performed in slices from long and short day animals, and allowed to compare rhythm amplitudes in both groups with an equal number of action potentials that contributed to the recording over the same time interval (c.f. Figure 4). The thresholds were determined so that each trigger level includes 10^5^ more spikes than the previous level. For all experiments the deviation from the aimed number of action potentials selected for was <5%.

Statistical analyses were performed in Origin 7 (OriginLab Corporation) and Excel (Microsoft). All values are stated as average±standard error of the mean (s.e.m.). Whenever the calculated value is the result of a difference between groups, such as in the calculation of in vitro phase shifts, variances were considered unequal, rendering a conservative test. P-values were calculated with a two sided t test and were considered to be significant when p<0.05.

## Supporting Information

Figure S1 Short and long photoperiod PRCs obtained from simulations (A) The distributions for short and long day subpopulations were taken from VanderLeest et al., 2007 [6]. Each vertical line represents the peak time of a small subpopulation of neurons. (B) A fitted curve through the long and short day length distribution was used to distribute 100 single unit PRCs. The y-axis represents differently phased single unit PRCs, distributed according to the fitted curve. The blue part of each line represents the delay part of the single unit PRC, the red part represents the advance part of the single unit PRC. The left side shows the distribution for short days and the right side shows the distribution for long days. (C) The resulting simulated ensemble PRC for short and long days using type 1 single unit PRCs. The long day PRC shows a lower amplitude than the short day PRC. (D) The area under the curve of the PRC decreases exponentially when the phase distribution of the neurons increases. The area is given relative to the area under the curve when all single unit PRCs coincide, which leads to a maximum amplitude of the PRC of the ensemble, and a maximal working area. On the x-axis, the observed distributions for the short and long day lengths are indicated. The figure shows the results for type 1 single unit PRCs. The results indicate that the area under the curve for short days is about two times larger than for long days, consistent with experimental results (see also Figure S3).
(0.49 MB TF

Figure S2 Magnitude of the light induced phase shift (A) Experimentally obtained subpopulation distributions [6] were used to distribute 100 type 1 single unit PRCs. The simulations resulted in high amplitude phase shifts for short days and low amplitude shifts for long days. Short day shifts were normalized to 100% and long day shifts were plotted relative to this value. (B) The same procedure was followed for type 0 single unit phase response curves. (C) For comparison, the experimentally obtained phase shifts in running wheel activity are depicted with the shift in short days normalized to 100% (p<0.001).
(0.04 MB PDF)

Figure S3 Area under the phase response curve (A) Quantitative analysis of the PRC based on type 1 single unit PRCs by a measurement of the area under the curve. For short days, the area was normalized to 100% and for long days, the area was plotted as a fraction of this value. (B) The same procedure was repeated for type 0 single unit PRCs. (C) The relative area under the curve from experimentally obtained behavioral PRCs. The area under the PRC in long day length is 45% of the normalized area in short day length.
(0.04 MB PDF)

Text S1 Supplemental Data and Figures
(0.06 MB DOC)
