# Supplementary figures and images for: Phase Shifting Capacity of the Circadian Pacemaker Determined by the SCN Neuronal Network Organization
Source: PLoS One. 2009 Mar 23;4(3):e4976. doi: 10.1371/journal.pone.0004976 (PMC2655235; doi:10.1371/journal.pone.0004976)

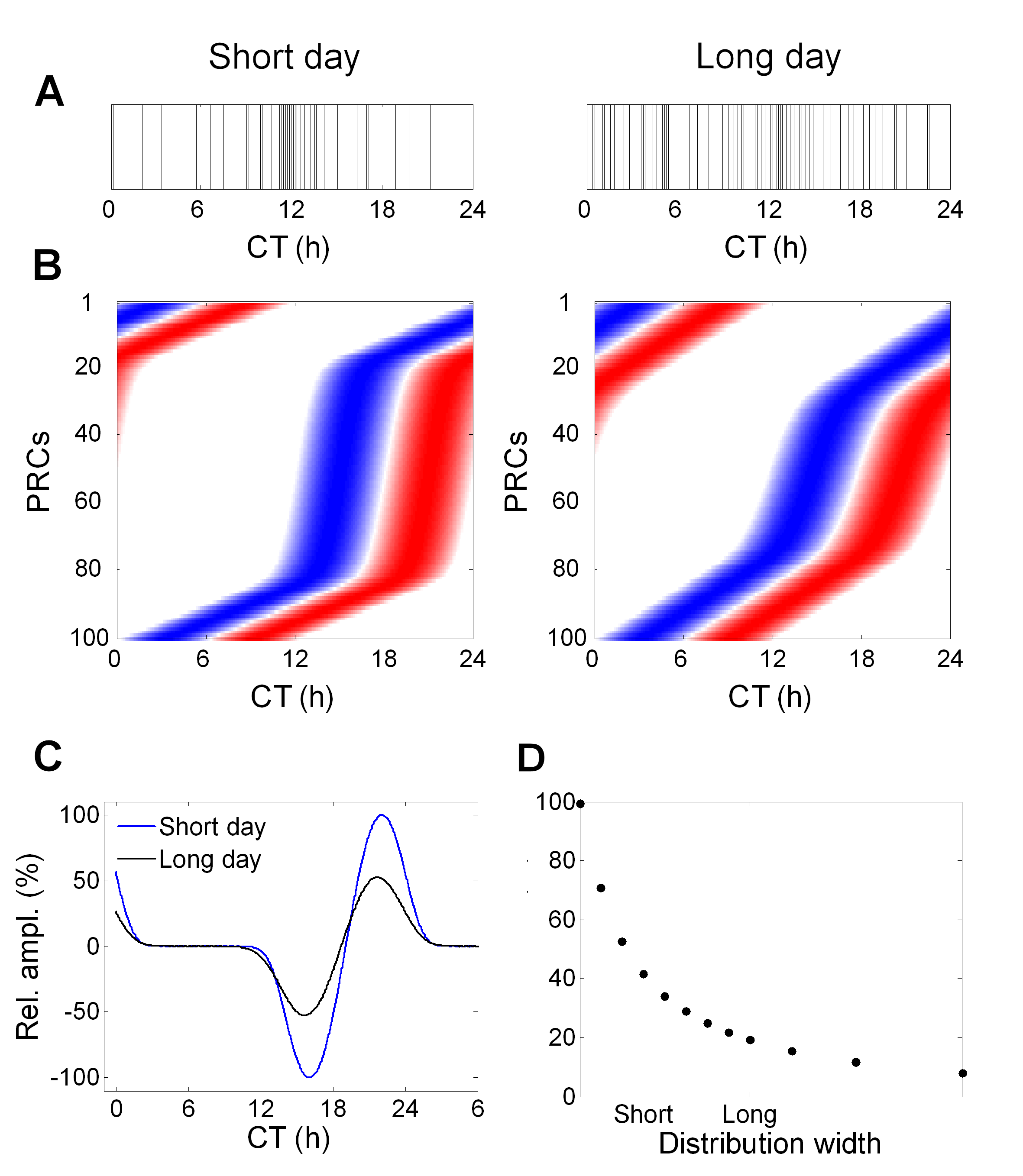

Supplement: Figure S1 — Short and long photoperiod PRCs obtained from simulations (A) The distributions for short and long day subpopulations were taken from VanderLeest et al., 2007 [6]. Each vertical line represents the peak time of a small subpopulation of neurons. (B) A fitted curve through the long and short day length distribution was used to distribute 100 single unit PRCs. The y-axis represents differently phased single unit PRCs, distributed according to the fitted curve. The blue part of each line represents the delay part of the single unit PRC, the red part represents the advance part of the single unit PRC. The left side shows the distribution for short days and the right side shows the distribution for long days. (C) The resulting simulated ensemble PRC for short and long days using type 1 single unit PRCs. The long day PRC shows a lower amplitude than the short day PRC. (D) The area under the curve of the PRC decreases exponentially when the phase distribution of the neurons increases. The area is given relative to the area under the curve when all single unit PRCs coincide, which leads to a maximum amplitude of the PRC of the ensemble, and a maximal working area. On the x-axis, the observed distributions for the short and long day lengths are indicated. The figure shows the results for type 1 single unit PRCs. The results indicate that the area under the curve for short days is about two times larger than for long days, consistent with experimental results (see also Figure S3). (0.49 MB TF [file pone.0004976.s001.tif]

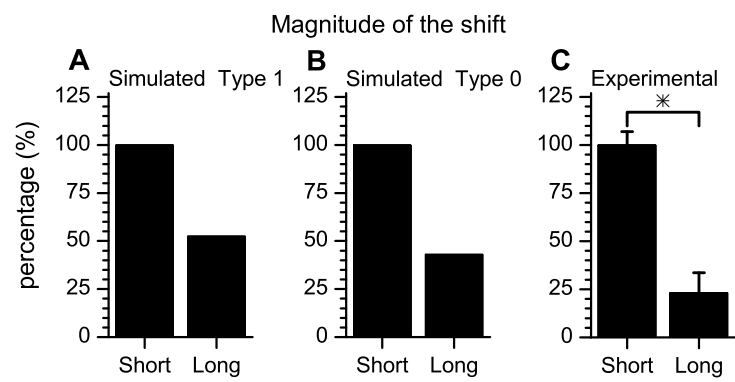

Supplement: Figure S2 — Magnitude of the light induced phase shift (A) Experimentally obtained subpopulation distributions [6] were used to distribute 100 type 1 single unit PRCs. The simulations resulted in high amplitude phase shifts for short days and low amplitude shifts for long days. Short day shifts were normalized to 100% and long day shifts were plotted relative to this value. (B) The same procedure was followed for type 0 single unit phase response curves. (C) For comparison, the experimentally obtained phase shifts in running wheel activity are depicted with the shift in short days normalized to 100% (p<0.001). (0.04 MB PDF) [file pone.0004976.s002.pdf]

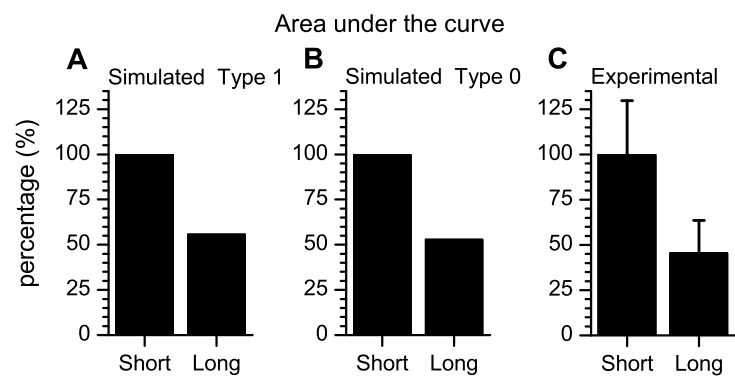

Supplement: Figure S3 — Area under the phase response curve (A) Quantitative analysis of the PRC based on type 1 single unit PRCs by a measurement of the area under the curve. For short days, the area was normalized to 100% and for long days, the area was plotted as a fraction of this value. (B) The same procedure was repeated for type 0 single unit PRCs. (C) The relative area under the curve from experimentally obtained behavioral PRCs. The area under the PRC in long day length is 45% of the normalized area in short day length. (0.04 MB PDF) [file pone.0004976.s003.pdf]
