# Supplementary material for: Phase Shifting Capacity of the Circadian Pacemaker Determined by the SCN Neuronal Network Organization
Source: PLoS One. 2009 Mar 23;4(3):e4976. doi: 10.1371/journal.pone.0004976 (PMC2655235; doi:10.1371/journal.pone.0004976)
Supplement: Text S1 — Supplemental Data and Figures (0.06 MB DOC) [file pone.0004976.s004.doc]

## Supplemental Data

### Simulations

Both molecular and electrophysiological studies have provided evidence that photoperiodically induced waveform changes observed at the population level [1,2] are caused by a reconfiguration of single cell activity patterns [3–7]. In short day length single units oscillate highly in phase while in long days they are more spread out over the circadian cycle. Because in short days the phase distribution among neurons is narrow, light information will reach SCN neurons at a similar phase of their cycle. When the distribution is broad, however, light information reaches neurons at different phases of their cycle. We performed simulations both with type 1 and with type 0 PRCs in the distributions. When distributing 100 type 1 PRCs, the amplitude for the long day length PRC is 52.5% of the amplitude for the short day length PRC; for type 0 PRCs, this ratio is 43% (Figure S2). The simulations revealed that irrespective of the type of single unit PRC, a broad distribution of cellular oscillations, corresponding to long days, results in a low amplitude PRC of the ensemble, and a narrow distribution, corresponding to short days, results in a high amplitude PRC of the ensemble (Figure S1). These results were independent of the number of single unit PRCs that were used in the simulations, although small deviations occurred for low numbers (n < 40). The simulated differences in the magnitude of the shifts resembled the experimentally obtained data (Figure S2).

We have also measured the area under the delay and advance part of the PRC for long and short day length PRCs for both the simulations and the experimentally obtained data (Figure S3). The area under the simulated long day PRC curve is about 50% of the area under the short day PRC. This was true for both types of single unit PRCs that were used to construct the ensemble PRC. For type 1 single unit PRCs, the area under the curve of the simulated long day PRC was 55.9% of the area under the curve of the short day PRC. For type 0 single unit PRCs, the area under the curve of the simulated long day PRC was 53% of the short day PRC. The results from these simulations were independent of the number of single unit PRCs and in accordance with the experimentally obtained data (Figure S3).

Simulation Methods

Simulations of a PRC for short day length and long day length were performed in MATLAB by distributing 100 normalized single unit phase response curves over the day. Two types of single unit PRCs were used. The first PRC consisted of a 12 h dead-zone (where no phase responses can be induced) and a 12 h sinusoidal responsive part, in accordance with the type 1 light pulse PRC [8]. The other single unit PRC was in accordance with a type 0 light pulse PRC, consisting of a 12 h dead-zone followed by an exponential function with an asymptote at CT18, and a maximum shift of 12 h [8]. The distributions that were used for long and short day lengths were taken directly from experimentally described subpopulation distributions in long and short photoperiods [6]. The peak times of these subpopulations were used to fit a distribution curve. This curve was used to distribute 100 single unit PRCs over the circadian day. In addition, simulations were performed using single unit PRCs without a dead-zone (c.f. Ukai et al., 2007 [9], data not shown).

We have measured the area under the curve, which is the surface of the delay and advance part of the PRC. The surface is an indication for the phase shifting capacity of the circadian system. The equation for this calculation is the first integral of a curve over a certain interval:$\int_{a}^{b} f(x)dx$ or $\sum\left|y\right|\times\Delta x$ for discrete functions, where the absolute value of y is multiplied by the width on the x-axis.

## Supplemental Figure Legends

### Figure S1

Short and long photoperiod PRCs obtained from simulations

(A) The distributions for short and long day subpopulations were taken from VanderLeest et al., 2007 [6]. Each vertical line represents the peak time of a small subpopulation of neurons. (B) A fitted curve through the long and short day length distribution was used to distribute 100 single unit PRCs. The y-axis represents differently phased single unit PRCs, distributed according to the fitted curve. The blue part of each line represents the delay part of the single unit PRC, the red part represents the advance part of the single unit PRC. The left side shows the distribution for short days and the right side shows the distribution for long days. (C) The resulting simulated ensemble PRC for short and long days using type 1 single unit PRCs. The long day PRC shows a lower amplitude than the short day PRC. (D) The area under the curve of the PRC decreases exponentially when the phase distribution of the neurons increases. The area is given relative to the area under the curve when all single unit PRCs coincide, which leads to a maximum amplitude of the PRC of the ensemble, and a maximal working area. On the x-axis, the observed distributions for the short and long day lengths are indicated. The figure shows the results for type 1 single unit PRCs. The results indicate that the area under the curve for short days is about two times larger than for long days, consistent with experimental results (see also Figure S3).

### Figure S2

Magnitude of the light induced phase shift

(A) Experimentally obtained subpopulation distributions [6] were used to distribute 100 type 1 single unit PRCs. The simulations resulted in high amplitude phase shifts for short days and low amplitude shifts for long days. Short day shifts were normalized to 100% and long day shifts were plotted relative to this value. (B) The same procedure was followed for type 0 single unit phase response curves. (C) For comparison, the experimentally obtained phase shifts in running wheel activity are depicted with the shift in short days normalized to 100% (p < 0.001).

### Figure S3

Area under the phase response curve

(A) Quantitative analysis of the PRC based on type 1 single unit PRCs by a measurement of the area under the curve. For short days, the area was normalized to 100% and for long days, the area was plotted as a fraction of this value. (B) The same procedure was repeated for type 0 single unit PRCs. (C) The relative area under the curve from experimentally obtained behavioral PRCs. The area under the PRC in long day length is 45% of the normalized area in short day length.

Supplemental References

1. Mrugala M, Zlomanczuk P, Jagota A, Schwartz WJ (2000) Rhythmic multiunit neural activity in slices of hamster suprachiasmatic nucleus reflect prior photoperiod. Am J Physiol Regul Integr Comp Physiol 278: R987-R994.

2. Sumova A, Jac M, Sladek M, Sauman I, Illnerova H (2003) Clock gene daily profiles and their phase relationship in the rat suprachiasmatic nucleus are affected by photoperiod. J Biol Rhythms 18: 134-144.

3. Schaap J, Albus H, VanderLeest HT, Eilers PH, Detari L, Meijer JH (2003) Heterogeneity of rhythmic suprachiasmatic nucleus neurons: Implications for circadian waveform and photoperiodic encoding. Proc Natl Acad Sci U S A 100: 15994-15999.

4. Hazlerigg DG, Ebling FJ, Johnston JD (2005) Photoperiod differentially regulates gene expression rhythms in the rostral and caudal SCN. Curr Biol 15: R449-R450.

5. Inagaki N, Honma S, Ono D, Tanahashi Y, Honma K (2007) Separate oscillating cell groups in mouse suprachiasmatic nucleus couple photoperiodically to the onset and end of daily activity. Proc Natl Acad Sci U S A 104: 7664-7669.

6. VanderLeest HT, Houben T, Michel S, Deboer T, Albus H, Vansteensel MJ, Block GD, Meijer JH (2007) Seasonal encoding by the circadian pacemaker of the SCN. Curr Biol 17: 468-473.

7. Naito E, Watanabe T, Tei H, Yoshimura T, Ebihara S (2008) Reorganization of the suprachiasmatic nucleus coding for day length. J Biol Rhythms 23: 140-149.

8. Johnson CH (1999) Forty years of PRCs--what have we learned? Chronobiol Int 16: 711-743.

9. Ukai H, Kobayashi TJ, Nagano M, Masumoto KH, Sujino M, Kondo T, Yagita K, Shigeyoshi Y, Ueda HR (2007) Melanopsin-dependent photo-perturbation reveals desynchronization underlying the singularity of mammalian circadian clocks. Nat Cell Biol 9: 1327-1334.
